# mTOR, autophagy, and reprogramming

**DOI:** 10.3389/fcell.2013.00004

**Published:** 2014-01-16

**Authors:** Bor Luen Tang

**Affiliations:** Department of Biochemistry, Yong Loo Lin School of Medicine, National University Health System, NUS Graduate School for Integrative Sciences and Engineering, National University of SingaporeSingapore, Singapore

**Keywords:** mTOR, autophagy, iPSC reprogramming, yamanaka factors, SOX2

Nuclear reprogramming to achieve induced-pluripotency by the Yamanaka factors (Takahashi and Yamanaka, 2006) is largely viewed as a consequence of a cascade of expression profile changes, along with alterations in epigenetic markings, which are primarily nuclear events. Cytoplasmic processes that could be critical for this process have not been emphasized. However, Fan and colleagues now showed that macroautophagy (or simply autophagy), regulated through the suppression of mTOR expression by Sox2, is required for nuclear reprogramming and induced pluripotent stem cell (iPSC) formation (Wang et al., 2013). These findings are in general agreement with two earlier reports. The first of which had shown that the mTOR inhibitor rapamycin enhanced iPSC generation (Chen et al., 2011), while the other demonstrated that reprogramming requires delicate modulation of mTOR levels and activity (He et al., 2012).

The authors has an elegant system of fibroblast harboring the Yamanaka factors (or OKSM factors—Oct4, Klf4, Sox-2, and c-Myc) that are doxycycline inducible. They observed that reprogramming to iPSCs is impaired in fibroblasts depleted of critical autophagy factors, such as Atg3, Atg5, and Atg7. There is an increase in the rate of autophagy, as indicated by the accumulation of LC3 puncta, within a day after doxycycline addition, and this peaked in day 2. The levels of the Atg proteins are not altered during iPSC induction, but mechanistic target of rapamycin (mTOR) transcript and protein levels, as well as readouts of its kinase activity, declined at the earlier stage of induction. The timing of this mTOR suppression is important, as the mTOR inhibitor rapamycin enhanced iPSC formation when administered at day 1 of induction, but inhibited iPSC formation when given 3 days after. Of the four Yamanaka factors, only ectopic expression of Sox2 could suppress mTOR expression. Interestingly, this occurs in fibroblasts, but not seen with cell lines such as HeLa or HEK293. The authors' observation has an important physiological correlate in early embryonic development, as they observed that autophagy is also induced in the 4–8 cell stage of developing embryos.

How does Sox2 suppress mTOR expression during the early stage of iPSC induction? The authors identified a Sox2 binding site at a region about 1.6 kbp upstream of the transcription start site of the mTOR gene. Deletion of this site using TALEN-based genome editing abolished Sox2 suppression of mTOR and autophagy induction, and the mutated cells could not generate iPSCs. That Sox2 suppression of mTOR expression at an early stage of induction is critical for iPSC production is further corroborated by the demonstration that inducible silencing of mTOR at the early stage bypassed the need for Sox2. The authors showed further that Sox2 act by recruiting the nucleosome remodeling deacetylase (NuRD) complex (Hu and Wade, 2012) to the repressor cis element of mTOR's promoter. Silencing of NuRD components restored mTOR expression, impairing autophagy induction and iPSC generation. The association of the repressor complex with mTOR promoter is dynamic, and the association is lost 3 days after iPSC induction. Again the physiological correlation between the *in vitro* fibroblast system and 4–8 cell stage embryos holds true, as NuRD is also found to be recruited to the mTOR promoter of embryos at this stage.

The findings of Fan and colleagues highlighted a crucial role for autophagy during nuclear reprogramming, and deciphered the underlying mechanism of how timely onset of autophagy during reprogramming is regulated through mTOR suppression by Sox2-NuRD. This regulatory mechanism is likely to be cell type dependent, or at least limited to untransformed cells. Another recent report has shown that iPSC-like cells generated by reprogramming of MCF-7 breast cancer cells have high expression of endogenous Sox2 (Corominas-Faja et al., 2013). This was, however, associated with transcriptional suppression of mTOR repressors, and consequentially an increased mTOR activity. Whether there was a decrease in mTOR activity at the very early stages of MCF-7 iPSC induction was unclear.

mTOR is a focal regulatory point of cellular homeostasis, and are linked to multiple signaling pathways that might impact on reprogramming. However it does appear that autophagy induced by mTOR suppression *per se* is critical for nuclear reprogramming in culture. Autophagy is known to be essential for early, preimplantation embryonic development, and Atg5-deficient embryos could not develop beyond the 4–8 cell stage (Tsukamoto et al., 2008). These findings beg the questions of why and how does autophagy augment reprogramming *in vitro* and *in vivo*. There are several possibilities. In drawing parallels between the transformation of fibroblasts to iPSCs with differentiation of precursor cells to adipocytes or erythrocytes, Vessoni and colleagues have postulated that extensive cellular remodeling in the cytoplasm is required in the establishment of induced pluripotency (Vessoni et al., 2012). One such remodeling involves reduction of mitochondria number, as iPSCs, like embryonic stem cells (ESCs), have reduced mitochondrial mass and reactive oxygen species (ROS) production (Armstrong et al., 2010; Prigione et al., 2010). As iPSCs are supposed to be rejuvenated from cumulative cellular and genetic damages found in its precursors, autophagy is an obvious mechanism whereby this rejuvenation could conceivably be achieved. Materials scavenged from autophagy could also provide anabolic building blocks and energy that would be channeled toward cellular remodeling. Autophagy-dependent cellular remodeling is conceivably also required during early embryonic development. This notion, however, does not address why mTOR suppression and autophagy induction is required at the very early stages of iPSC induction.

Another possible connection is the potential ability of autophagy to enhance cell survival and alleviate apoptosis and/or senescence. Menendez and colleagues have argued that “forced expression of the Yamanaka cocktail of stemness factors is a stressful process that activates apoptosis and cellular senescence” (Menendez et al., 2011). The latter processes are mediated by the tumor suppressor p53 and its downstream effectors, which are known barriers to reprogramming (Hong et al., 2009; Marión et al., 2009; Li et al., 2013). p53-mediated inhibition of reprogramming was postulated as a mechanism that might ensure genomic integrity of the pluripotent stem cells produced by programming. It appears that a parallel process occurs in the cytoplasm to ensure integrity of the structural and functional proteome in reprogrammed stem cells. There are tantalizing clues that these two processes crosstalk with each other. Identification of the points of connection between them would be of tremendous academic interest, and will undoubtedly also facilitate the efficacy and quality of iPSC production *in vitro*.
